# A 17 year-old girl with a demyelinating disease requiring mechanical ventilation: a case report

**DOI:** 10.1186/1756-0500-6-22

**Published:** 2013-01-18

**Authors:** Chrysostomos Katsenos, Despoina Androulaki, Stavroula Lyra, Theodoros Tsoutsouras, Costas Mandragos

**Affiliations:** 1 Korgialenio-Benakio Red Cross Hospital of Athens, ICU, Athanasaki 1 str, Ampelokipoi, 11526, Athens, Greece; 2 Korgialenio-Benakio Red Cross Hospital of Athens, MRI UNIT, Athanasaki 1 str, Ampelokipoi, 11526, Athens, Greece

## Abstract

**Background:**

Demyelinating diseases cause destruction of the myelin sheath, while axons are relatively spared. Pathologically, demyelination can be the result of an inflammatory process, viral infection, acquired metabolic derangement and ischemic insult. Three diseases that can cause inflammatory demyelination of the CNS are: Multiple sclerosis (MS), Acute disseminated encephalomyelitis (ADEM) and Acute hemorrhagic leucoencephalitis. Differentiation is not always easy and there is considerable overlaping. Data about adults with acute demyelination requiring ICU admission is limited.

**Case presentation:**

A 17 year old Greek female was hospitalised in the ICU because of acute respiratory failure requiring mechanical ventilation. She had a history of febrile disease one month before, acute onset of paraplegia, diplopia, progressive arm weakness and dyspnea. Her consciousness was not impaired. A demyelinating central nervous system (CNS) disease, possibly post infectious encephalomyelitis (ADEM) was the underlying condition. The MRI of the brain disclosed diffused expanded cerebral lesions involving the optic nerve, basal ganglia cerebellum, pons and medulla oblongata. There was also extended involvement of the cervical and thoracic part of the spinal cord. CSF leukocyte count was elevated with lymphocyte predominance. The patient required mechanical ventilation for two months. Then she was transferred to a rehabilitation centre. Three years later she remains paraplegic. Since then she has not suffered any other demyelination attack.

**Conclusions:**

Demyelinating diseases can cause acute respiratory failure when the spinal cord is affected. Severe forms of these diseases, making necessary ICU admission, is less frequently reported. Intensivists should be aware of the features of these rare diseases.

## Background

The term demyelination describes a destruction of the myelin sheath. Axons that have been demyelinated, secondarily, tend to atrophy and degenerate. Demyelination according to pathogenesis can be classified into several categories: inflammatory, viral, metabolic, hypoxic-ischemic and compressive [1].

Inflammatory demyelination occurs in multiple sclerosis (MS), acute disseminated encephalomyelitis (ADEM) and acute hemorrhagic leucoencephalitis (AHL). MS can be classified, according to clinical and pathological features, into three main subtypes: classical, acute and concentric sclerosis. Devic’s disease (neuromyelitis optica) is correlated with a specific autoantibody (NMO-IgG). It was considered a variant of MS, but recent pathological, immunopathogenic and imaging studies suggest distinctive features [2]. Although there are specific pathological features associated with these diseases, differential diagnosis is mainly based on clinical and medical imaging grounds. In addition, connective tissue diseases, bacterial and viral infections and other conditions, can cause transverse myelopathy.

Excluding Devic’s disease, there is no biological marker that can help differentiate these diseases. Clinical features depend on the region of the CNS that is affected and on the extent of demyelination. Generally, MS is more common in adults, tends to relapse, and is usually monosymptomatic. While ADEM is more common in children, frequently accompanying encephalopathy with seizures. It is not relapsing and follows a history of precipitating infection within two weeks. Optic neuritis is unilateral in MS and bilateral in ADEM. The CSF examination commonly shows oligoclonal IgG in MS and lymphocytosis with increased protein in ADEM, while the MRI shows disseminated inflammatory lesions, which are periventricular or callosal in MS and cortical or in deep gray matter in ADEM.

When the spinal cord is involved in ADEM, lesions are large, swollen and thoracic, whereas in MS they are clearly smaller, more discrete and cervical. The follow up can distinguish the two diseases. In ADEM new lesions should not occur and original lesions are expected to resolve partially or completely. If a new lesion occurs after the acute/subacute phase, a diagnosis of MS is more probable. In many cases, however, the final diagnosis can only be made after a considerably long period and follow up examinations [3-7]. Although ADEM in adults has been studied, there is limited data of severe forms requiring ICU admission. Common causes for referral to ICU are seizures, impaired consciousness and fever. Prognosis is poor and survivors have functional impairments [8].

## Case presentation

A 17 year old Greek female was hospitalised in the ICU because of acute respiratory failure requiring mechanical ventilation. A degenerative central nervous system (CNS) disease, possibly post infectious encephalomyelitis (ADEM) was the underlying condition. Upon hospital admission the patient complained of headache, low back pain and fever. Within days, the patient progressively deteriorated with muscle weakness in the upper and lower extremities.

**Figure 1:**
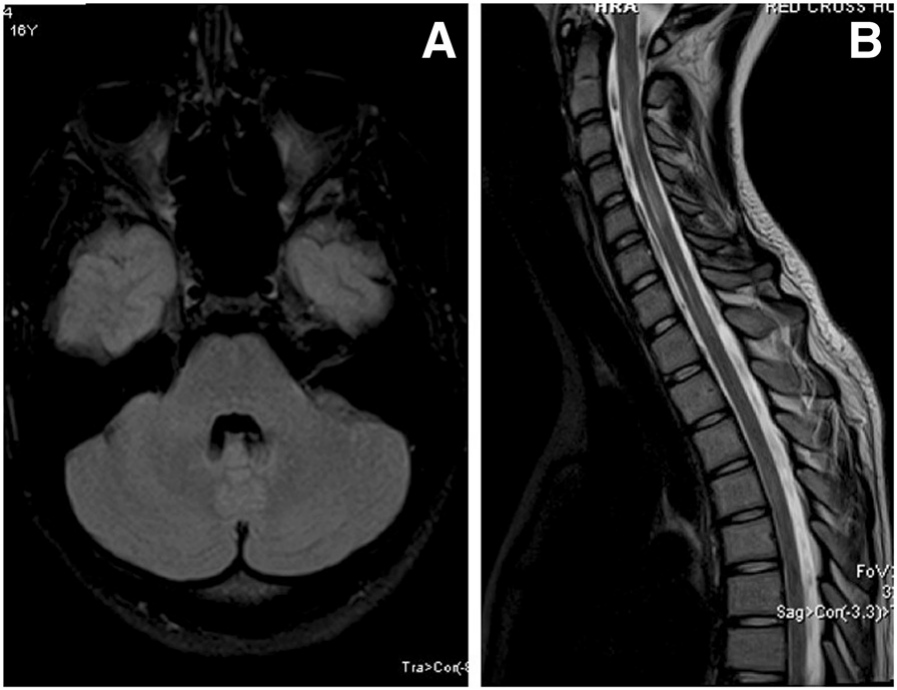
**A. MRI Flair axial of the brain; normal. B**: T2 sagital cervical and upper thoracic spine; intramedullary lesions of cervical and thoracic spine C3 –Th5.

The MRI of the brain was normal (Figure 1A), but the cervical and upper thoracic MRI revealed intramedullary inflammatory lesions extending from C3 to Th5 (Figure 1B). The patient was treated with high doses of corticosteroids (methylprednisolone 1gr per day for 4 days), with no response. Gradually, visual disturbances developed and the patient complained of dyspnea. An oxygen saturation of 85% was observed by pulse oxymetry, and oxygen administration (Venturi mask 50%) followed. Her condition rapidly deteriorated. The patient was intubated immediately, supported with mechanical ventilation and transferred to the ICU. (No spirometric evaluation or blood gas analysis was performed).

**Figure 2:**
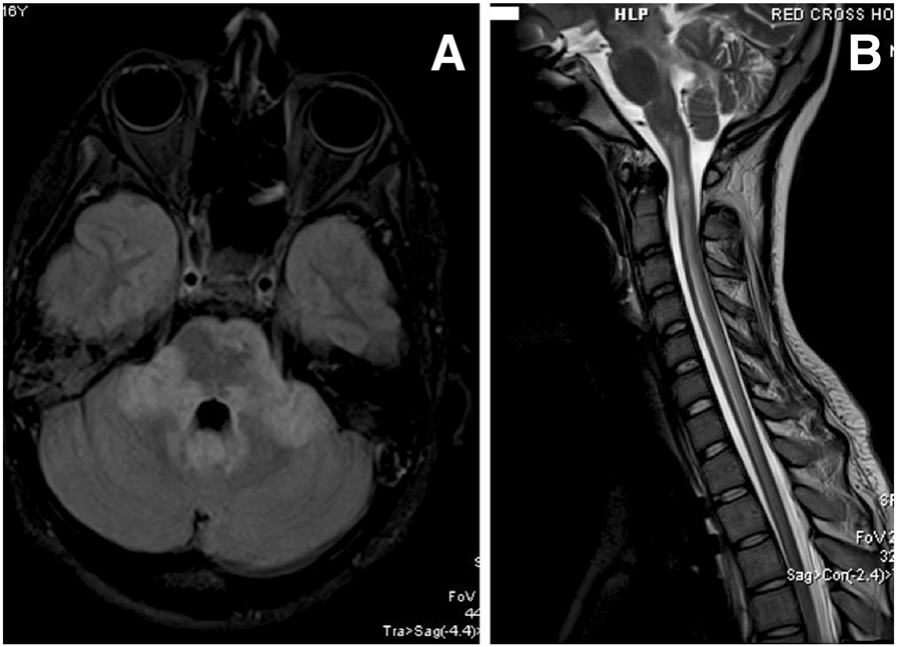
**A. Flair axial of the brain; extensive lesions mainly in brain stem and cerebellum. B**: Spine T2 sagital. Extensive confluent lesions extending cranially up to the medulla oblongata and caudally to the middle thoracic spine.

Intravenous immune globulin (IVIG) treatment was administered (30 grams per day for 4 days). An MRI of the brain revealed diffused extensive lesions, mainly in brain stem and cerebellum, with a high T2 signal compatible with myelitis (Figure 2A). The MRI of the spine revealed extensive confluent lesions extending cranially up to the medulla oblongata and caudally to the middle of the thoracic spine (Figure 2B). CSF was abnormal (WBC = 240/ml, mainly lymphocytes, total protein = 750 mg/dl, glucose ratio CSF/serum = 45/92 mg/dl and no oligoclonal bands of IgG). CSF PCR examination was negative for HSV1,2, enteroviruses, n. meningitis, streptococcus pneumonia, listeria, haemophilus influenza, TBC. In the ICU no additional data was found by repeated cerebral MRI. Neutropenia developed and bone marrow examination was performed, which disclosed a normal bone marrow. The neutropenia probably was due to neutrophil’s peripheral destruction, as the patient suffered from severe sepsis, due to ventilator associated pneumonia. All attempts to liberate the patient from the ventilator failed, as the patient could not use the intercostal muscles. A new CSF examination showed normal, serum NMO-IgG, ANA and ACE were negative. The patient underwent tracheostomy and gastrostomy.

**Figure 3:**
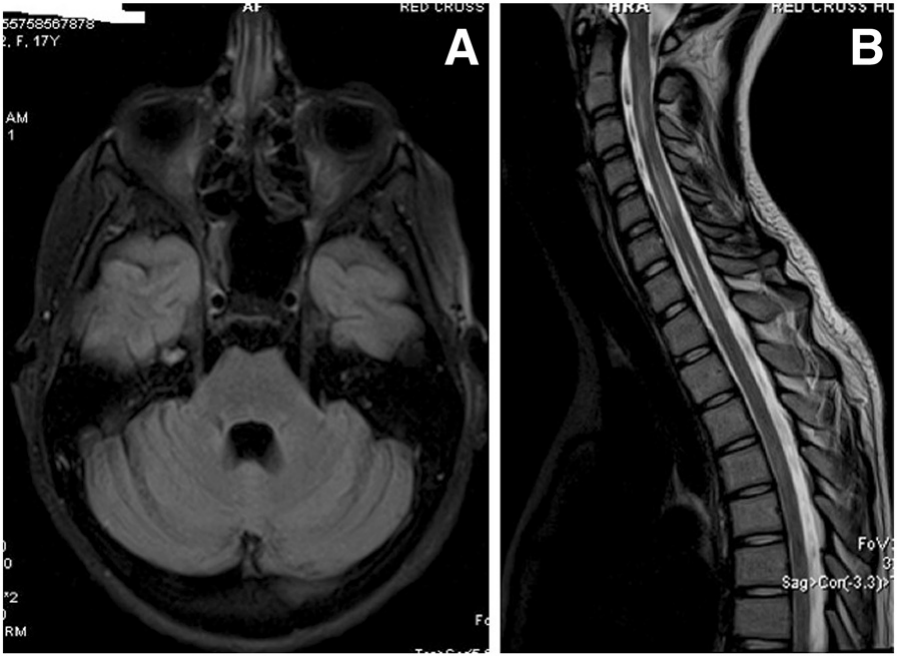
**A. Flair axial brain; normal after four months. B**: Cervical spine T2 sagital. After four months, most of the intramedullary lesions have been disappeared.

A month later, the patient’s condition gradually improved, allowing her exemption from the ventilator. A new MRI of the brain was normal (Figure 3A) and most of the intramedullary lesions had vanished (Figure 3B). Her consciousness level improved. Some motion was observed in her arms. Although blindness remained in her right eye, gradual improvement allowed closure of tracheostomy and gastrostomy. However she could not move her lower extremities and had no sense of touch. During the last weeks of hospitalisation she had a persistent bacteriuria by multidrug resistant gram(−) bacteria. She also experienced a right pneumothorax, conservatively treated with success.

Three years after discharge the patient is still paraplegic despite the resolution of the lesions in MRI. She complains of a bilateral loss in her senses of heat and touch at the level of the thighs.

## Discussion

Differential diagnosis between demyelinating diseases is not easy due to a lack of specific biological markers. In our case, the history of a febrile disease a month before the development of neurological symptoms supports the diagnosis of ADEM. Our patient did not suffer any demyelination attack during the three years of follow up. This is also supportive to the diagnosis of ADEM, which is monophasic illness as opposed to relapsing-remitting MS. Contrary to this diagnosis is the fact that patient’s consciousness remained intact while she had no seizures. Devic’s disease was ruled out by the absence of the NMO-IgG.

A possible cause of the acute respiratory failure is the extensive spinal cord involvement, as the patient under clinical examination displayed prolonged intercostal muscle weakness. Unfortunately respiratory function testing was not done before intubation and the respiratory failure was not well studied.

A persistent paraplegia along with a loss of senses in heat and touch despite MRI normalisation is another interesting point worthy of observation. One possible explanation is the development of critical illness polyneuropathy (CIP) and myopathy (CIM) during the prolonged ICU stay. These two clinical entities may occur concomitantly and are associated with sepsis, multiorgan failure and possibly with corticosteroid treatment [9].

Our patient was treated with high doses of corticosteroids and suffered many times from sepsis and severe sepsis during her long ICU hospitalisation. Unfortunately, she was not available for nerve conduction studies and electromyography.

Another explanation is that the traditional MRI in MS and in other demyelinating diseases has a poor correlation with clinical disability. The MRI can detect changes in inflammatory activity but for quantification of intact myelin and remyelination, magnetization transfer imaging has been proposed. Diffusion tensor imaging can be used to monitor tract-specific changes that may be more closely linked to clinical measures and may be particularly powerful when combined with functional measures, such as functional MRI [10,11].

## Conclusion

Severe forms of demyelinating diseases, requiring ICU admission, bear a poor prognosis. Clinical recovery may be limited despite resolution of imaging changes.

## Consent

“Written informed consent was obtained from the patient, who is now 20 years old, for publication of this case report and accompanying images. A copy of the written consent is available for review by the Editor-in-Chief of this journal.”

## Abbreviations

MRI: Magnetic resonance imaging; ADEM: Acute disseminated encephalomyelitis; MS: Multiple sclerosis; CSF: Cerebrospinal fluid; ANA: Antinuclear antibodies; ACE: Angiotensin converting enzyme; CIP: Critical illness polyneuropathy; CIM: Critical illness myopathy.

## Competing interests

The authors declare that they have no competing interests.

## Authors’ contributions

CK, cared for the patient and co-ordinated the presentation. DA reviewed the laboratory test results and prepared the case presentation. SL, reviewed the MRI and provided the legends. TT did the follow up of the patient and contributed in writing. CM cared for the patient and supervised the writing of the manuscript. All authors read and approved the final manuscript.
